# A subpopulation of human bone marrow erythroid cells displays a myeloid gene expression signature similar to that of classic monocytes

**DOI:** 10.1371/journal.pone.0305816

**Published:** 2024-07-22

**Authors:** Roman Perik-Zavodskii, Olga Perik-Zavodskaia, Julia Shevchenko, Marina Volynets, Saleh Alrhmoun, Kirill Nazarov, Vera Denisova, Sergey Sennikov

**Affiliations:** 1 Laboratory of Molecular Immunology, Federal State Budgetary Scientific Institution Research Institute of Fundamental and Clinical Immunology, Novosibirsk, Russia; 2 Department of Natural Sciences, Novosibirsk State University, Novosibirsk, Russia; 3 Clinic of Immunopathology, Federal State Budgetary Scientific Institution Research Institute of Fundamental and Clinical Immunology, Novosibirsk, Russia; European Institute of Oncology, ITALY

## Abstract

Erythroid cells, serving as progenitors and precursors to erythrocytes responsible for oxygen transport, were shown to exhibit an immunosuppressive and immunoregulatory phenotype. Previous investigations from our research group have revealed an antimicrobial gene expression profile within murine bone marrow erythroid cells which suggested a role for erythroid cells in innate immunity. In the present study, we focused on elucidating the characteristics of human bone marrow erythroid cells through comprehensive analyses, including NanoString gene expression profiling utilizing the Immune Response V2 panel, a BioPlex examination of chemokine and TGF-beta family proteins secretion, and analysis of publicly available single-cell RNA-seq data. Our findings demonstrate that an erythroid cell subpopulation manifests a myeloid-like gene expression signature comprised of antibacterial immunity and neutrophil chemotaxis genes which suggests an involvement of human erythroid cells in the innate immunity. Furthermore, we found that human erythroid cells secreted CCL22, CCL24, CXCL5, CXCL8, and MIF chemokines. The ability of human erythroid cells to express these chemokines might facilitate the restriction of immune cells in the bone marrow under normal conditions or contribute to the ability of erythroid cells to induce local immunosuppression by recruiting immune cells in their immediate vicinity in case of extramedullary hematopoiesis.

## Introduction

Erythroid cells, formerly deemed as neglected cells [1], have recently garnered increased attention from researchers owing to their discernible immunoregulatory functionalities. Extensive investigations involving both human and murine erythroid cells, facilitated by techniques such as flow cytometry, bulk RNA-seq, and qPCR, have elucidated their capacity to generate and express a diverse array of immunosuppressive molecules. Noteworthy among these are interleukin-10 (IL-10) [2, 3] and transforming growth factor-beta 1 (TGF-beta1) [4, 5] cytokines, as well as the production of reactive oxygen species (ROS) [6, 7]. Additionally, erythroid cells have been found to express programmed cell death ligand-1 (PD-L1) [5], PD-L2 [6], and V-domain Ig suppressor of T cell activation (VISTA) [8] inhibitory checkpoint molecules, along with the enzymatic activities of Arginase-1 and Arginase-2 [5, 9–11].

While some researchers have posited the immunoregulatory properties of erythroid cells based on their synthesis of a diverse repertoire of multidirectional cytokines with both pro- and anti-inflammatory attributes [12–17], it is noteworthy that existing data is contradictory in nature. Variability arises particularly in delineating the precise mechanisms employed by erythroid cells to exert their immunoregulatory influence.

In our preceding investigation [4], we identified an antibacterial gene expression signature in murine bone marrow erythroid cells, wherein the genes *S100a8*, *S100a9*, and *Camp* exhibited the highest overall expression levels. This myeloid cell-like gene expression signature in murine erythroid cells raised the question of whether such genes are also expressed in human erythroid cells.

In a recent study of human erythroid cells [18], a novel population of cells termed erythroid-derived myeloid cells (EDMCs) was characterized as "transcriptionally indistinguishable from their myeloid-derived counterparts." These cells emerge in cancer patients from CD45-positive erythroid cells, induced by tumor-derived granulocyte-macrophage colony-stimulating factor (GM-CSF). This phenomenon adds another dimension to the versatile nature of erythroid cells, demonstrating myeloid properties, particularly those associated with immunosuppression, albeit in a non-steady state.

Presently, the transferrin receptor CD71 stands as the predominant marker employed for the study of erythroid cells. This marker has been described as a marker for the enrichment of erythroid cells, yielding an average purity of 85–95% [10]. The resulting enriched cell population is commonly referred to as CD71+ erythroid cells or CECs. However, it is imperative to exercise caution when applying this enrichment method under conditions associated with T-cell activation [19, 20] and/or acute lymphoid and myeloid leukemia, as these cells may also express CD71, greatly contaminating the isolated erythroid cell population [21].

Other erythroid cell markers, that are truly specific to erythroid cells are sialoglycoproteins Glycophorin A (CD235a) [22] and Glycophorin B (CD235b) [23], and ALAS2 –an erythroid-specific heme synthesis enzyme [24]. Mitoferrin 1 (*SLC25A37* gene)–an iron-transporting protein, essential for heme synthesis [25], can also be used as a marker of erythroid cells, albeit it is not erythroid-specific and can only be treated as an enrichment marker among other more specific markers like ALAS2.

**Fig 1:**
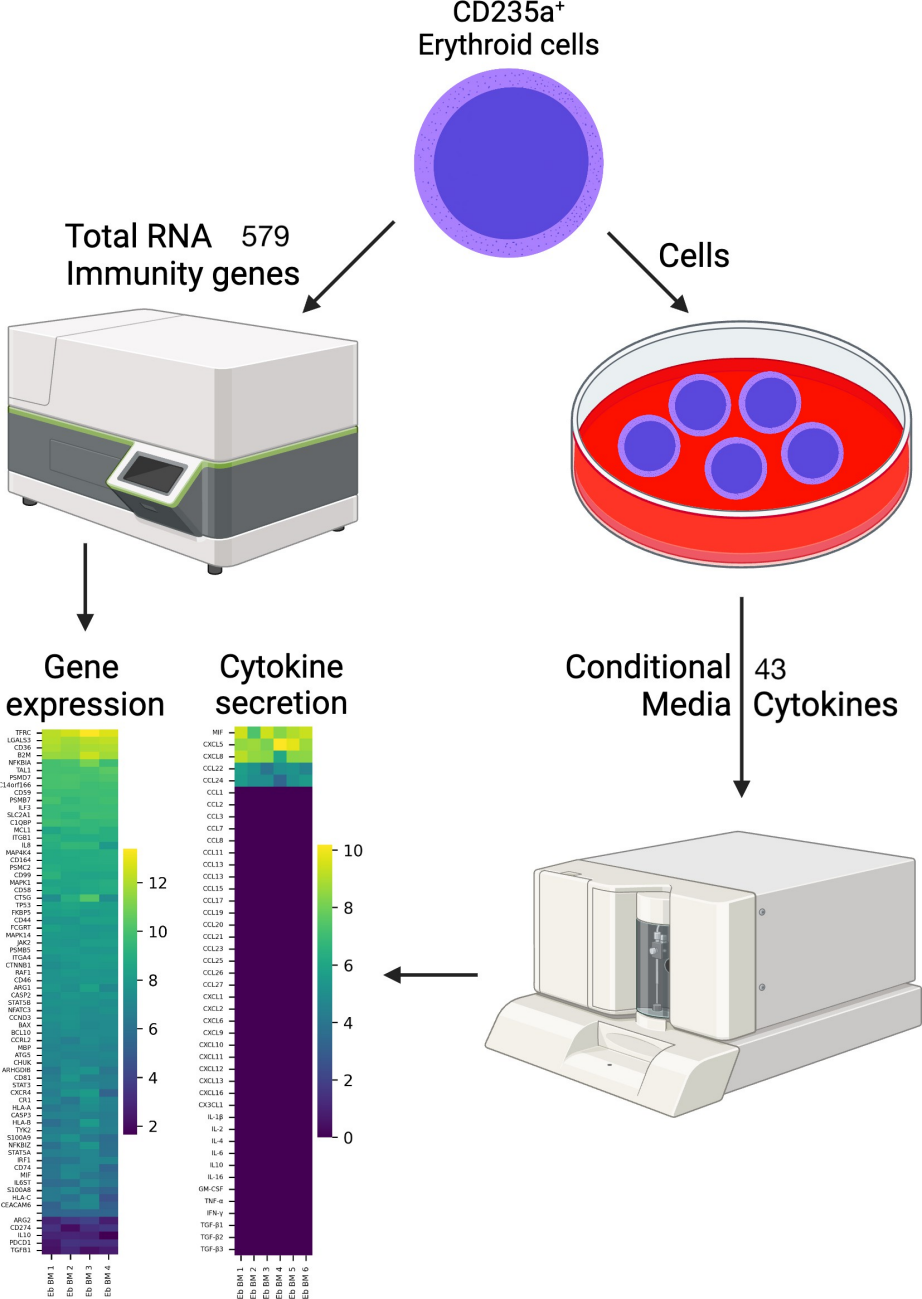
Overview of the experiment. This figure was created via BioRender.

In this investigation, we undertook a comprehensive analysis of the transcriptome of healthy adult human CD235a-positive erythroid cells (Erythroid cells) utilizing the NanoString method for bulk RNA profiling, conducted an in-depth analysis of our previously published CD235a-positive adult healthy human bone marrow erythroid cell single-cell RNA sequencing (scRNA-seq) data, employing modern single-cell analysis, as well as probed the secretion of cytokines, chemokines, and TGF-beta proteins of Erythroid cells using the Bio-Plex platform (Fig 1).

We aimed to unveil the spectrum of genes encoding for the immunoregulatory cytokines, enzymes, and surface proteins expressed by healthy adult human Erythroid cells.

## Results

### Human bone marrow Erythroid cells display a Myeloid-like gene expression signature

We performed a bulk immune transcriptome study of healthy adult human bone marrow Erythroid cells obtained by positive magnetic sorting for CD235a via the NanoString method (*n* = 4) to study the involvement of such cells in immunity. Genes with detected expression (sorted in the descending mean detected probe count order) were: *TFRC*, *LGALS3*, *CD36*, *B2M*, *NFKBIA*, *TAL1*, *PSMD7*, *C14orf166*, *CD59*, *PSMB7*, *ILF3*, *SLC2A1*, *C1QBP*, *MCL1*, *ITGB1*, *IL8 (CXCL8)*, *MAP4K4*, *CD164*, *PSMC2*, *CD99*, *MAPK1*, *CD58*, *CTSG*, *TP53*, *FKBP5*, *CD44*, *FCGRT*, *MAPK14*, *JAK2*, *PSMB5*, *ITGA4*, *CTNNB1*, *RAF1*, *CD46*, *ARG1*, *CASP2*, *STAT5B*, *NFATC3*, *CCND3*, *BAX*, *BCL10*, *CCRL2*, *MBP*, *ATG5*, *CHUK*, *ARHGDIB*, *CD81*, *STAT3*, *CXCR4*, *CR1*, *HLA-A*, *CASP3*, *HLA-B*, *TYK2*, *S100A9*, *NFKBIZ*, *STAT5A*, *IRF1*, *CD74*, *MIF*, *IL6ST*, *S100A8*, *HLA-C* and *CEACAM6*.

**Fig 2:**
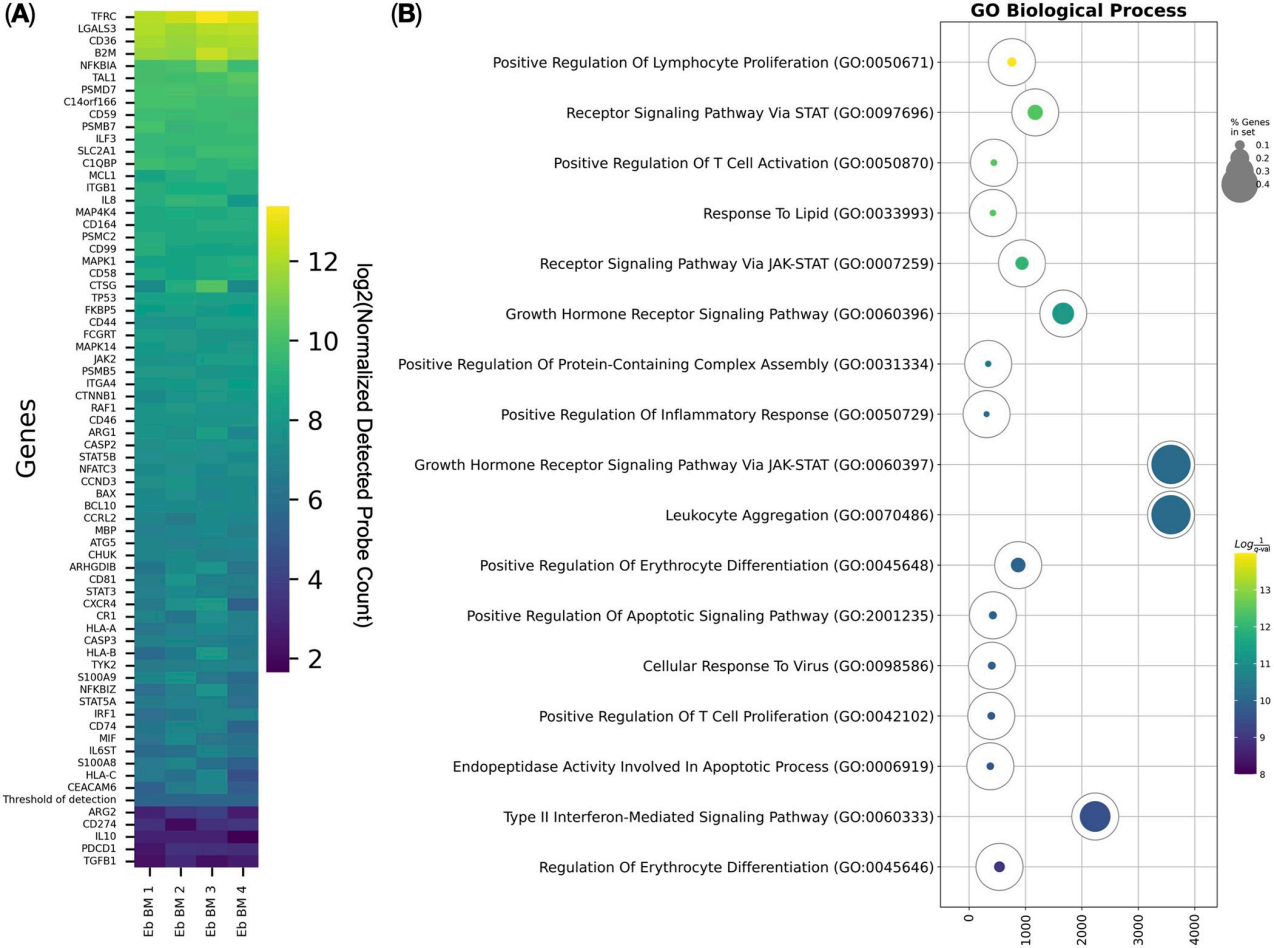
Immunity-related gene expression by Erythroid cells. (A) Heatmap of NanoString Human Immunology V2 panel genes expressed by the adult human bone marrow Erythroid cells (Eb BM). The heatmap shows log2-transformed values of the mean detected probe count, sorted in descending order; the yellow color corresponds to the maximum expression, and the deep purple color corresponds to the absence of expression (*n* = 4). (B) Gene Ontology Biological Process overrepresentation analysis of the genes with the detected expression in human adult bone marrow Erythroid cells. The yellow color corresponds to the lowest *q*-value, the deep purple color corresponds to the highest *q*-value, and the fullness of the bubble reflects the percentage of genes in the analysis from the full set of genes in the Gene Ontology Biological Process database.

The most abundant expression-wise genes show a conservative erythroid signature: *TFRC*, *LGALS3*, *CD36*, and *TAL1* [4, 9] which marks the high purity of the cells (Fig 2A).

We only detected *IL8* (*CXCL8*) and *MIF* chemokine gene expression among all the cytokines and chemokines included in the NanoString V2 Immunology panel.

*ARG2* (Arginase 2), *CD274* (PD-L1), *IL10* (IL-10), *PDCD1* (PD-1), and *TGFB1* (TGF-β1) gene expression was not detected.

We then performed Gene Ontology Biological Process overrepresentation analysis on the NanoString Human Immunology V2 panel genes with the detected expression in human adult bone marrow Erythroid cells and observed a “Response To Lipid” gene expression signature in the adult human bone marrow Erythroid cells (Fig 2B, Table 1).

**Table 1:** Gene Ontology Biological Process overrepresentation analysis of the genes with the detected expression in human adult bone marrow Erythroid cells.

Gene Ontology Biological Process Term	Overlap	*Q*-value	Score	Genes
Positive Regulation Of Lymphocyte Proliferation	7/74	0,0000	759	*CD74*, *TFRC*, *CD81*, *TYK2*, *IL6ST*, *CD46*, *JAK2*
Positive Regulation Of T Cell Activation	7/107	0,0000	441	*TFRC*, *CD81*, *TYK2*, *IL6ST*, *CD46*, *JAK2*, *B2M*
Receptor Signaling Pathway Via STAT	5/31	0,0000	1173	*STAT5A*, *STAT5B*, *STAT3*, *TYK2*, *JAK2*
Response To Lipid	7/110	0,0000	424	*STAT5B*, *STAT3*, *CTNNB1*, *CD36*, *JAK2*, *S100A9*, *S100A8*
Receptor Signaling Pathway Via JAK-STAT	5/36	0,0000	938	*STAT5A*, *STAT5B*, *STAT3*, *TYK2*, *JAK2*
Growth Hormone Receptor Signaling Pathway	4/17	0,0000	1670	*STAT5A*, *STAT5B*, *STAT3*, *JAK2*
Positive Regulation Of Protein-Containing Complex Assembly	6/98	0,0000	340	*LGALS3*, *TFRC*, *TAL1*, *BAX*, *CD36*, *TP53*
Positive Regulation Of Inflammatory Response	6/104	0,0000	311	*NFKBIA*, *CD81*, *NFKBIZ*, *IL6ST*, *S100A9*, *S100A8*
Growth Hormone Receptor Signaling Pathway Via JAK-STAT	3/7	0,0000	3578	*STAT5A*, *STAT5B*, *STAT3*
Leukocyte Aggregation	3/7	0,0000	3578	*S100A9*, *CD44*, *S100A8*
Positive Regulation Of Erythrocyte Differentiation	4/26	0,0000	871	*STAT5B*, *TAL1*, *STAT3*, *MAPK14*
Positive Regulation Of Apoptotic Signaling Pathway	5/62	0,0000	423	*BAX*, *BCL10*, *S100A9*, *TP53*, *S100A8*
Cellular Response To Virus	5/64	0,0001	404	*CHUK*, *BAX*, *TYK2*, *MAPK14*, *JAK2*
Positive Regulation Of T Cell Proliferation	5/65	0,0001	395	*TFRC*, *TYK2*, *IL6ST*, *CD46*, *JAK2*
Endopeptidase Activity Involved In Apoptotic Process	5/67	0,0001	378	*BAX*, *CASP2*, *JAK2*, *S100A9*, *S100A8*
Type II Interferon-Mediated Signaling Pathway	3/9	0,0001	2236	*IRF1*, *TYK2*, *JAK2*
Regulation Of Erythrocyte Differentiation	4/36	0,0001	540	*STAT5B*, *TAL1*, *STAT3*, *MAPK14*

This signature was comprised of *STAT5B*, *STAT3*, *CTNNB1*, *CD36*, *JAK2*, *S100A8*, and *S100A9* genes. Among these genes, *S100A8* and *S100A9*, antimicrobial immunity genes, were of particular interest. These genes were the dominant genes expression-wise in murine erythroid cells profiled with a similar NanoString Mouse Immunology V1 panel [4]. But, unlike in murine erythroid cells, *S100A8* and *S100A9* gene expression were not as high in adult human bone marrow Erythroid cells, which suggests that only a fraction of adult human bone marrow Erythroid cells could express these genes.

### A subpopulation of Erythroid cells displays a myeloid gene expression signature similar to that of classic monocytes

**Fig 3:**
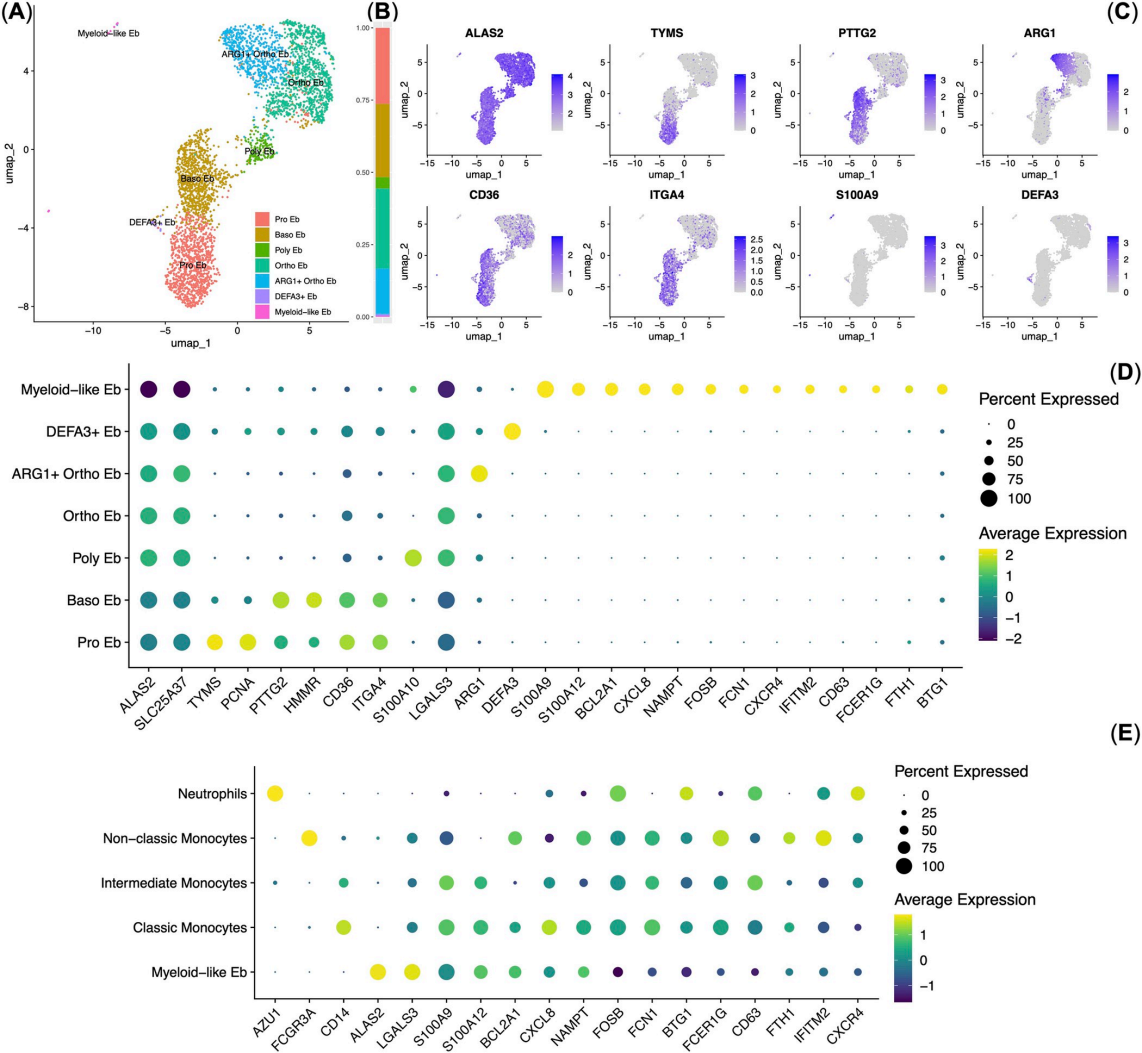
Analysis of the Erythroid cell single-cell RNA sequencing data (*n* = 3). (**A)** UMAP plot of the clusters, each cluster is color-labelled; **(B)** stacked bar plot of the percentages of Erythroid cells per cluster; **(C)** Feature plots of the Erythroid cell cluster-defining genes, the grey color represents the absence of the marker expression whereas the deep blue color represents the maximum of the marker expression; **(D)** dot plot that shows cluster-specific gene expression signatures, mean marker expression values were Z-score transformed, the deep purple color represents the lowest marker expression whereas the yellow color represents the maximum of the marker expression, dot size represents the percentage of Erythroid cells positive for the marker; **(E)** dot plot that shows Myeloid-like Erythroid cell cluster-specific gene expression signature in Myeloid cells (*n* = 4) and Myeloid-like Erythroid cells (*n* = 3), mean marker expression values were Z-score transformed, the deep purple color represents the lowest marker expression whereas the yellow color represents the maximum of the marker expression, dot size represents the percentage of erythroid cells positive for the marker.

Next, we decided to perform an advanced analysis of our previously published healthy adult human bone marrow Erythroid cell single-cell RNA sequencing (scRNA-seq) data [9]. The scRNA-seq data (*n* = 3) was generated using Erythroid cells from the same 3 out of 4 donors in the aforementioned NanoString analysis and using the same separation method (CD235a-positive magnetic separation) and had high gene expression correlation with the NanoString data (R = 0.8). We performed UMAP dimensionality reduction and clustering of Erythroid cells and found all stages of erythroid cell differentiation: proerythroblasts (Pro Eb), basophilic erythroblasts (Baso Eb), polychromatophilic erythroblasts (Poly Eb), orthochromatophilic erythroblasts (Ortho Eb), as well as *ARG1* gene expressing orthochromatophilic erythroblasts (*ARG1+* Ortho Eb) and two previously undetected subpopulations– *DEFA3*+ Erythroid cells (DEFA3+ Eb) and the population that we predicted from the bulk NanoString data—*S100A9* expressing Erythroid cells that we termed Myeloid-like Erythroid cells (Myeloid-like Eb) (*S100A8* gene was not included in the scRNA-seq panel) (Fig 3A and 3C).

*DEFA3*+ Erythroid cells comprised 2% of the bone marrow Erythroid cells while Myeloid-like Erythroid cells comprised 1% of the bone marrow Erythroid cells (Fig 3B).

Both *DEFA3*+ Erythroid cells and Myeloid-like Erythroid cells had the gene expression of the hallmark erythroid gene *ALAS2* [24] and erythroid-enriched gene *SLC25A37* [25] and had no gene expression of any myeloid marker present in the scRNA-seq panel (*AZU1*, *CD14*, *FCGR3A* (CD16)) [26, 27]. They also had the subpopulation-unique *DEFA3* (*DEFA3*+ Erythroid cells) and *BCL2A1*, *NAMPT*, *S100A12*, *IFITM2*, *FTH1*, *BTG1*, *CXCR4*, *CXCL8*, *CD63*, *DUSP1*, *FCER1G*, *S100A9*, *FOSB*, and *FCN1* (Myeloid-like Erythroid cells) gene expression respectively (Fig 3D and 3E).

The expression of the *CD274* (PD-L1), *IL10*, *PDCD1* (PD-1), and *TGFB1* genes was not detected in the adult human bone marrow Erythroid cell scRNA-seq data, which is in accord with the NanoString data (See Fig 2). The *ARG2* gene was not included in the scRNA-seq panel.

We then compared the gene expression of Myeloid-like Erythroid cells with other myeloid cells–Classic, Intermediate, and Non-classic monocytes and Neutrophils from the healthy adult bone marrow that we obtained from the pre-clustered publicly-available scRNA-seq data and found that Myeloid-like Erythroid cell gene expression signature resembles that of Classic monocytes the most among myeloid cells (Fig 3E) and that Myeloid-like Erythroid cells express *IFITM2*, *BCL2A1*, *CXCR4*, *NAMPT*, *S100A12*, *FTH1*, *BTG1*, *CD63*, *CXCL8*, *HLA-A*, *DUSP1*, *FCER1G*, *S100A9*, *FOSB*, and *GAPDH* genes on the same level as Classic monocytes (Table 2).

**Table 2:** Differential gene expression analysis data between Myeloid-like Erythroid cells and Classic monocytes via *FindMarkers*.

Genes	Log2(Fold Change)	% Cells Expressing in Myeloid-like Eb	% Cells Expressing in Classic Monocytes	*q*-value
***SNCA***	8.37	100.0	0.0	0.0000000
***ALAS2***	7.76	100.0	0.0	0.0000000
***SLC25A37***	7.27	100.0	0.0	0.0000000
***YBX3***	5.12	100.0	20.0	0.0000000
***LGALS3***	4.15	100.0	25.3	0.0000000
*IFITM2*	2.25	50.0	24.6	0.1034076
*BCL2A1*	1.88	75.0	38.2	1.0000000
*CXCR4*	1.78	41.7	18.6	1.0000000
*NAMPT*	1.77	66.7	73.8	1.0000000
*S100A12*	1.55	83.3	80.2	1.0000000
*FTH1*	1.39	41.7	19.8	1.0000000
*BTG1*	0.88	58.3	40.2	1.0000000
*CD63*	0.33	41.7	60.5	1.0000000
*CXCL8*	-0.22	66.7	87.1	1.0000000
*HLA-A*	-0.27	100.0	100.0	1.0000000
*DUSP1*	-0.60	75.0	90.3	1.0000000
*FCER1G*	-0.73	41.7	92.2	1.0000000
*S100A9*	-0.74	100.0	100.0	1.0000000
*FOSB*	-1.79	58.3	95.2	0.1027069
*GAPDH*	-1.85	50.0	92.4	0.0071662
***FCN1***	-2.55	50.0	100.0	0.0000160

Log2(Fold Change) threshold = 0.0, minimal cell percentage = 0.0%), genes with the 0% expression in Myeloid-like Erythroid cells were dropped from the analysis, differentially expressed (with *q*-value < 0.005) gene names are highlighted in bold.

**Fig 4:**
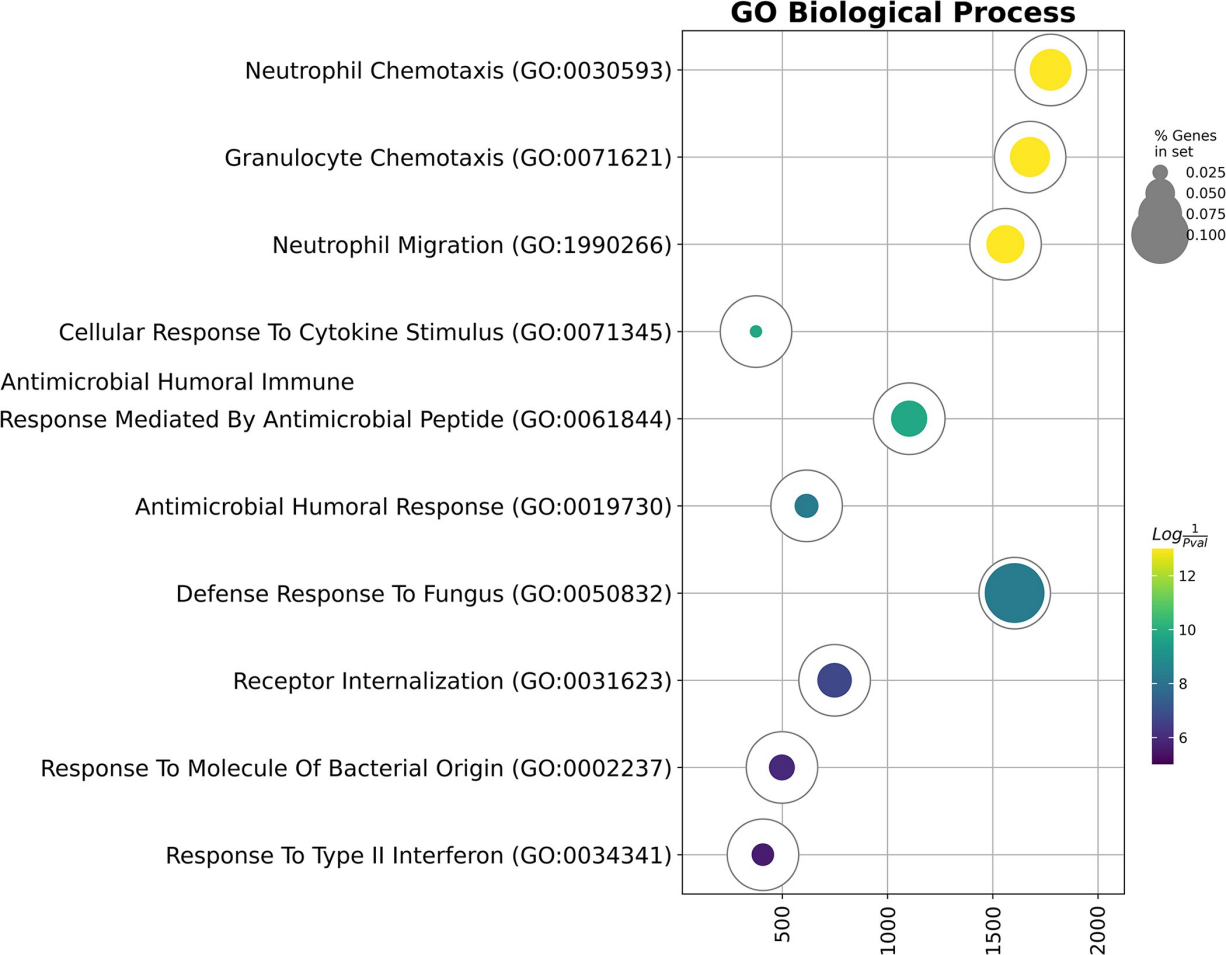
Gene Ontology Biological Process overrepresentation analysis of the genes with the detected expression in Myeloid-like Erythroid cells. The yellow color corresponds to the lowest *q*-value, and the deep purple color corresponds to the highest *q*-value, and the fullness of the bubble reflects the percentage of genes in the analysis from the full set of genes in the Gene Ontology Biological Process database.

We then performed Gene Ontology Biological Process overrepresentation analysis on the genes with the detected expression in Myeloid-like Erythroid cells–*ALAS2*, *SLC25A37*, *SNCA*, *YBX3*, *LGALS3*, *IFITM2*, *BCL2A1*, *CXCR4*, *NAMPT*, *S100A12*, *FTH1*, *BTG1*, *CD63*, *CXCL8*, *HLA-A*, *DUSP1*, *FCER1G*, *S100A9*, *FOSB*, *FCN1*, and *GAPDH* and observed an overrepresentation in the “Neutrophil Chemotaxis”, “Antimicrobial Humoral Response Mediated By Antimicrobial Peptide” and “Defense Response To Fungus” terms (Fig 4, Table 3).

**Table 3:** Gene Ontology Biological Process overrepresentation analysis of the genes expressed by the Myeloid-like Erythroid cells.

Gene Ontology Biological Process Term	Overlap	*Q*-value	Score	Genes
Neutrophil Chemotaxis	5/70	0,0000	1775	*LGALS3*, *CXCL8*, *FCER1G*, *S100A12*, *S100A9*
Granulocyte Chemotaxis	5/73	0,0000	1677	*LGALS3*, *CXCL8*, *FCER1G*, *S100A12*, *S100A9*
Neutrophil Migration	5/77	0,0000	1560	*LGALS3*, *CXCL8*, *FCER1G*, *S100A12*, *S100A9*
Cellular Response To Cytokine Stimulus	6/308	0,0000	375	*CXCL8*, *IFITM2*, *DUSP1*, *CXCR4*, *YBX3*, *GAPDH*
Antimicrobial Humoral Immune Response Mediated By Antimicrobial Peptide	4/65	0,0000	1103	*CXCL8*, *S100A12*, *S100A9*, *GAPDH*
Antimicrobial Humoral Response	4/100	0,0000	615	*CXCL8*, *S100A12*, *S100A9*, *GAPDH*
Defense Response To Fungus	3/29	0,0000	1604	*S100A12*, *S100A9*, *GAPDH*
Receptor Internalization	3/51	0,0000	748	*CXCL8*, *FCER1G*, *SNCA*
Response To Molecule Of Bacterial Origin	3/69	0,0000	498	*CXCL8*, *S100A9*, *SNCA*
Response To Type II Interferon	3/80	0,0000	408	*IFITM2*, *GAPDH*, *SNCA*

### Human bone marrow Erythroid cells secrete CCL22, CCL24, CXCL5, CXCL8, and MIF chemokines

Then, we studied the secretion of cytokines and chemokines in the 24h *in vitro*-cultured conditioned media from healthy adult human bone marrow Erythroid cells using the BioPlex mass secretomic method (*n* = 6). We also decided to confirm the absence of production and secretion of TGF-beta family proteins by the same method.

**Fig 5:**
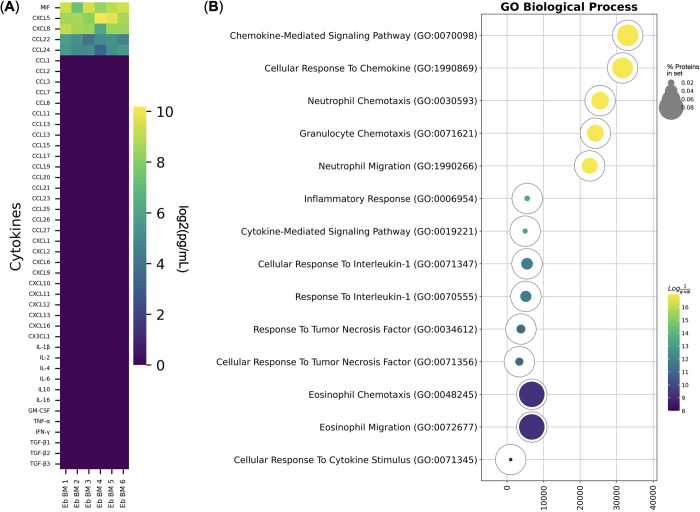
Cytokine secretion by Erythroid cells. (A) Heatmap of cytokines secreted by human bone marrow Erythroid cells (Eb BM). The heatmap shows log2-transformed values of cytokine concentrations in pg/mL, the yellow color corresponds to the maximum detected protein secretion, and the deep purple color corresponds to the absence of protein secretion (*n* = 6). (B) Gene Ontology Biological Process overrepresentation analysis of the cytokines with the detected secretion in human adult bone marrow Erythroid cells. The yellow color corresponds to the lowest *q*-value, and the deep purple color corresponds to the highest *q*-value, and the fullness of the bubble reflects the percentage of proteins in the analysis from the full set in the Gene Ontology Biological Process database.

We found secretion of the chemokines CCL22, CCL24, CXCL5, CXCL8, and MIF in the conditioned media from adult human bone marrow Erythroid cells. We have previously found *CXCL5* gene expression in the adult human bone marrow erythroid cells (this gene was not included in the NanoString Human Immunology panel) [9], *CXCL8* (*IL8*) and *MIF* gene expression was observed in the NanoString data (See Fig 1) and *CXCL8* (*IL8*) was unique to the Myeloid-like Erythroid cells (See Fig 3D). CCL22 and CCL24 genes were absent from both NanoString and scRNA-seq gene panels. We detected no production of IL10 and TGF-β1, TGF-β2, and TGF-β3 proteins, confirming the data from both transcriptomic analyses (Fig 5A).

We then performed Gene Ontology overrepresentation analysis of the detected chemokines—CCL22, CCL24, CXCL5, CXCL8, and MIF, and observed enrichment in the neutrophil and eosinophil chemotaxis Gene Ontology terms (Fig 5B, Table 4).

**Table 4:** Gene Ontology Biological Process overrepresentation analysis of the cytokines with the detected secretion by human adult bone marrow Erythroid cells.

Gene Ontology Biological Process Term	Overlap	Q-value	Score	Cytokines
Chemokine-Mediated Signaling Pathway	4/57	0,00000	33024	CCL24, CXCL8, CCL22, CXCL5
Cellular Response To Chemokine	4/59	0,00000	31614	CCL24, CXCL8, CCL22, CXCL5
Neutrophil Chemotaxis	4/70	0,00000	25485	CCL24, CXCL8, CCL22, CXCL5
Granulocyte Chemotaxis	4/73	0,00000	24176	CCL24, CXCL8, CCL22, CXCL5
Neutrophil Migration	4/77	0,00000	22609	CCL24, CXCL8, CCL22, CXCL5
Inflammatory Response	4/236	0,00000	5515	CCL24, CXCL8, CCL22, CXCL5
Cytokine-Mediated Signaling Pathway	4/257	0,00000	4945	CCL24, CXCL8, CCL22, CXCL5
Cellular Response To Interleukin-1	3/81	0,00001	5465	CCL24, CXCL8, CCL22
Response To Interleukin-1	3/85	0,00001	5144	CCL24, CXCL8, CCL22
Response To Tumor Necrosis Factor	3/108	0,00001	3807	CCL24, CXCL8, CCL22
Cellular Response To Tumor Necrosis Factor	3/119	0,00002	3369	CCL24, CXCL8, CCL22
Eosinophil Chemotaxis	2/24	0,00010	6775	CCL24, CCL22
Eosinophil Migration	2/24	0,00010	6775	CCL24, CCL22
Cellular Response To Cytokine Stimulus	3/308	0,00023	993	CCL24, CXCL8, CCL22

## Discussion

In this work, we performed transcriptomic and secretomic studies of adult human bone marrow Erythroid cells and were able to narrow down the spectrum of both expressed genes and secreted cytokines. We have found a gene expression signature in Erythroid cells using NanoString that included the antibacterial genes *S100A8* and *S100A9*, and a chemokine gene *CXCL8* (*IL8*), which we later mapped to a subpopulation of Erythroid cells we termed Myeloid-like Erythroid cells. These cells had detected expression levels of their genes similar to those of classic monocytes and gene expression signatures of antimicrobial immunity and neutrophil chemotaxis, which suggests Myeloid-like Erythroid cells as potential players in the antimicrobial immunity that could also recruit neutrophils to their site. This could be useful in case of bone marrow bacteremia, a highly lethal condition, that can happen during bone marrow transplantation [28]. Unlike erythroid-derived myeloid cells (EDMCs) [18], Myeloid-like Erythroid cells did not express any immunosuppressive genes like *ARG1*, *CD274* (PD-L1), *C10orf54* (VISTA), *PDCD1* (PD-1), *IL10*, or *TGFB1*, which suggests multiple different populations of erythroid cells with myeloid-like properties. Chemokine receptor *CXCR4* gene expression was unique to the Myeloid-like Erythroid cells which might restrict them to the bone marrow, as bone marrow stromal cells secrete CXCL12 –the ligand of CXCR4 [29], so we do not expect to observe Myeloid-like Erythroid cells outside of the bone marrow, therefore the expected antimicrobial protection function is only anticipated locally as well.

As all Myeloid-like Erythroid cells were positive for the *S100A9* gene and were CD235a-selected, a combination of *S100A9* gene expression and CD235a protein expression might be enough to find this population using the flow cytometry.

It is also puzzling to identify the stage of differentiation of Myeloid-like Erythroid cells. Myeloid-like Erythroid cells had very low levels of *ALAS2* and *SLC25A37* gene expression and their cluster was outside of the main branch of differentiation. This can either mean that these cells were at a very early stage of differentiation, or had left the main erythroid continuum.

As we have observed similar levels of *S100A8* and *S100A9* gene expression in our NanoString data, we suppose that the *S100A8* gene expressed in the Myeloid-like Erythroid cells as well and forms a *S100A8* / *S100A9* dimer–antimicrobial protein Calprotectin, as it was observed in murine erythroid cells [4].

In our Bio-Plex secretomic analysis of the healthy adult human Erythroid cell conditional media we have found the secretion of CCL22, CCL24, CXCL5, CXCL8, and MIF chemokines. This spectrum of chemokines could allow Erythroid cells to attract neutrophils and eosinophils, which could help Erythroid cells restrict the aforementioned cell types to the bone marrow in normal condition or initiate their migration to a site of extramedullary erythropoiesis. Murine erythroid cells also had gene expression of many chemokines (yet different—*Mif*, *Ccl2*, *Ccl3*, *Ccl9*, and *Cxcl12*) [4], which makes chemokine secretion feature evolutionary conservative among erythroid cells. Detected *CXCL8* gene expression was unique to the Myeloid-like Erythroid cells which means that this small subpopulation is solely responsible for all of the Erythroid cell production and secretion of CXCL8 (IL8).

We did not detect any IL10 gene expression in healthy adult bone marrow Erythroid cells as it was previously described [17]. This shows the importance of using newer methods for the validation purposes of some of the older studies and also means that healthy adult bone marrow Erythroid cells cannot cause IL-10-based immunosuppression in the bone marrow or at the site of extramedullary erythropoiesis.

Lack of the immunosuppressive *TGFB1* gene expression differentiates human Erythroid cells from murine erythroid cells, where *Tgfb1* was one the most expressed genes overall when profiled by the similar NanoString Mouse Immunology V1 panel [4], which suggests different immunosuppressive potential for human and mouse erythroid cells.

Healthy adult bone marrow Erythroid cells also differ from the tumor-induced Erythroid cells [6, 18], as they do not express any PDCD1 (PD-1) or CD274 (PD-L1) genes, which suggests restricted immunosuppression by healthy adult bone marrow Erythroid cells compared with the tumor-induced ones.

## Materials and methods

### Human bone marrow collection

We obtained bone marrow samples from both male (*n* = 3) and female (*n* = 3) healthy donors. The study subjects were between the ages of 23 and 28 without any underlying conditions and without any clinical evidence of anemia (*n* = 6). Bone marrow collection from healthy adult donors was approved by the local ethics committee of the Research Institute of Fundamental and Clinical Immunology at meeting No. 129, held in February 2021. We obtained written informed consent from all adult bone marrow donors involved in the study. The recruitment period for this study began on 01.03.2021 and ended on 30.11.2023.

### Cell isolation

We collected the bone marrow aspirates (up to 5 mL in volume) into tubes containing EDTA. We isolated bone marrow mononuclear cells using density gradient centrifugation (Ficoll-Paque^TM^ (Thermo Fisher Scientific, Waltham, MA, USA) with a density of 1.077 g/mL) at 266 RCF for 30 min to remove RBCs.

### Magnetic separation

We performed magnetic separation of the bone marrow mononuclear cells using a magnetic stand, a magnet (Miltenyi Biotec, 130-042-102, Bergisch Gladbach, Cologne, Germany), and CD235a MicroBeads (Miltenyi Biotec, 130-050-501, Bergisch Gladbach, Cologne, Germany) according to the manufacturer’s protocols.

### Viability staining

We measured the CD235 magnetically sorted erythroid cell viability on a Countess 3 Automated Cell Counter (Thermo Fisher Scientific, Waltham, MA, USA) according to the manufacturer’s protocols using trypan blue. Trypan blue staining showed >94% viability of the Erythroid cells.

### Flow cytometry data acquisition

We washed 2*10^6^ Erythroid cells in PBS containing 0.09% NaN_3_ and stained surface proteins with the BioLegend (San Diego, California, United States) antibodies: #334114 PerCP/Cyanine5.5 anti-human CD71 and #349104 FITC anti-human CD235a (Glycophorin A) antibodies according to the manufacturer’s protocols. We then washed the cells after 30 minutes of incubation in the dark with 0,5 ml PBS containing 0.09% NaN_3_. We then added BioLegend #423113 Zombie Violet™ to all samples.

**Fig 6:**
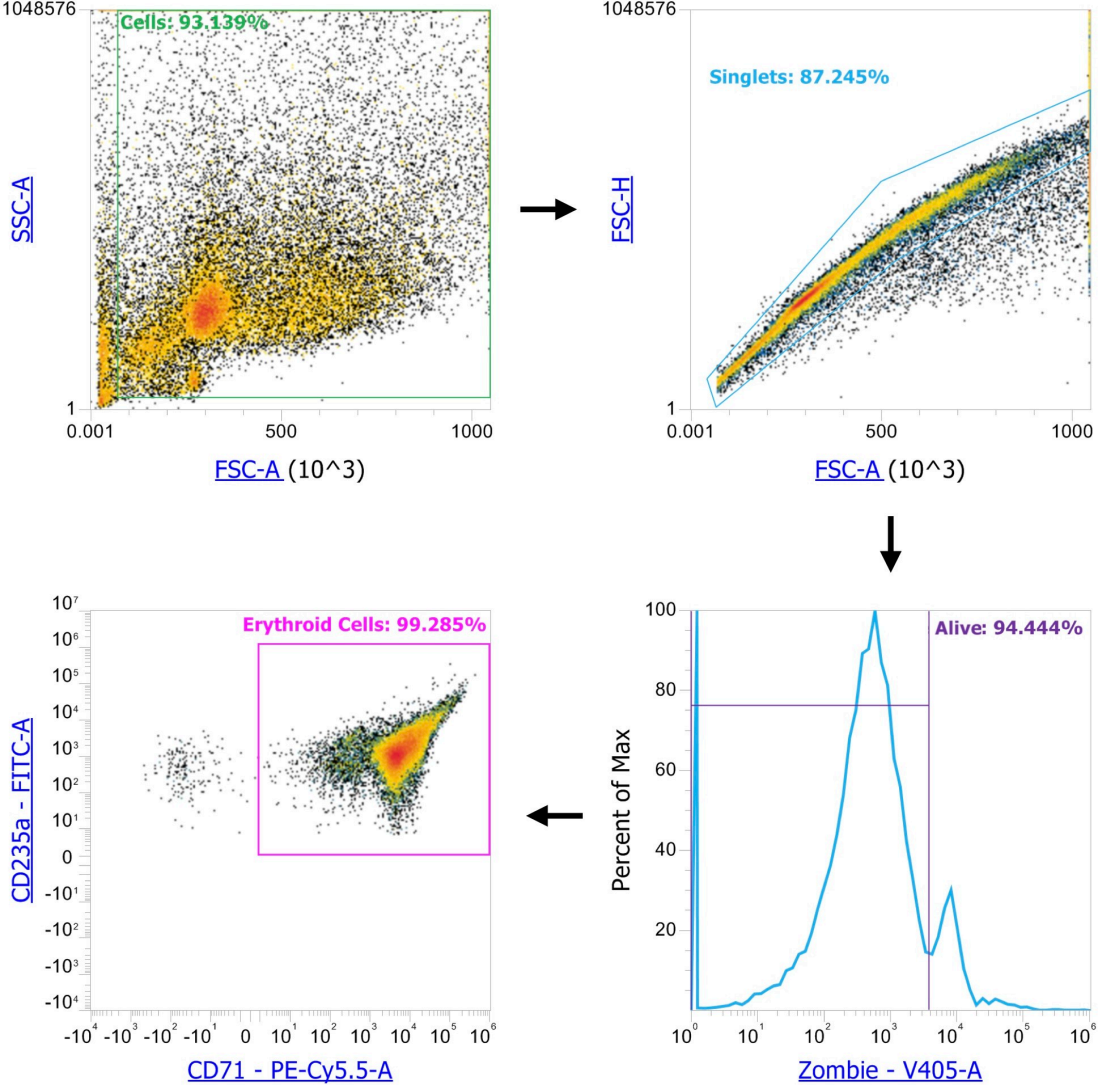
Erythroid cell gating strategy and purity assessment.

We manually gated cells from debris, singlets from the cells, alive cells from the singlets, and, finally, CD235-positive cells from the living cells in Attune NxT flow cytometer gating software. We observed > 99.0% purity of Erythroid cells (*n* = 6) and calculated cell count as events per uL (Fig 6).

### Cell culturing

We cultured the Erythroid cells in X-VIVO 10 serum-free medium (Lonza, Basel, Switzerland) with the addition of Insulin-Transferrin for 24 h at a concentration of 1 million per mL of the medium to support their viability and measure the culture medium’s cytokines afterward.

### Cell culture medium harvesting

We separated the Erythroid cell culture medium from the Erythroid cells after 24 h of culturing. We performed the separation by centrifugation at 1500 rpm for 10 min, transferred the cell culture medium into new 1.5 mL tubes with the addition of BSA up to a total concentration of 0.5%, and froze the cell culture medium at −80°C until the cytokine quantification.

### Cytokine quantification in culture medium

We used 50 μL of the bone marrow Erythroid cell (*n* = 6) culture media to quantify culture medium cytokines, chemokines and TGF-beta proteins in doubles using a Bio-Plex Pro™ Human Chemokine Panel, 40-Plex (BioRad, #171AK99MR2, Hercules, CA, USA), Bio-Plex Pro TGF-β 3-plex Assay (BioRad, #171W4001M, Hercules, CA, USA) on a Bio-Plex 200 instrument (BioRad, Hercules, CA, USA). We then log2-transformed the data frame, created a heatmap via bioinfokit [30] library for Python 3 of the cytokines, and performed gene ontology biological process overrepresentation analysis of the detected cytokines via GSEApy [31] library for Python 3.

### Total RNA extraction

We isolated total RNA from the bone marrow Erythroid cells (*n* = 4) after their magnetic separation and before culturing using a Total RNA Purification Plus Kit (Norgen Biotek, 48400, Thorold, Canada), measured the concentration of the RNA on a NanoDrop 2000c (Thermo Fisher Scientific, Waltham, USA) and diluted the RNA to a concentration of 10 ng/μL using nuclease-free water. We froze the diluted total RNA at −80°C until the immune transcriptome profiling.

### Immune transcriptome profiling by NanoString

We performed gene expression profiling with the help of the NanoString nCounter SPRINT Profiler analytical system using 100 ng of total RNA from each Erythroid cell sample (*n* = 4). We used a nCounter Human Immunology v2 panel to analyze the total RNA samples. The nCounter Human Immunology v2 panel consists of 579 immunity-associated genes, 15 housekeeping genes, and eight negative and six positive controls. The samples were subjected to a 20h hybridization reaction at 65°C, where 5–12 μL of total RNA was combined with 3 μL of nCounter Reporter probes, 0–7 μL of DEPC-treated water, 10 μL of hybridization buffer, and with 5 μL of nCounter capture probes (total reaction volume = 30 μL). After the 20h hybridization of the probes to targets of interest in the samples, the number of target molecules was determined on the NanoString nCounter SPRINT Profiler analytical system. We performed normalization and QC in nSolver 4 using added synthetic positive controls and the *RPL19*, *OAZ1*, *GAPDH*, *EEF1G*, *TUBB*, and *HPRT1* housekeeping genes included in the panel. We then performed background thresholding on the normalized data to remove non-expressing genes. The background level was determined as the mean of the POS_E controls and the genes that did not pass the threshold in at least one sample were removed. We manually added the threshold of detection (the mean of the POS_E controls) and *ARG2*, *CD274*, *IL10*, and *TGFB1* genes that were below the threshold of detection to the data frame. We then log2-transformed the data frame, created a heatmap via bioinfokit [30] library for Python 3 of the detected genes, and performed gene ontology biological process overrepresentation analysis of the detected genes via GSEApy [31] library for Python 3.

### Single-cell RNA-seq data analysis via Seurat

We analyzed our previously published publicly-available adult bone marrow CD235a-positive erythroid cell scRNA-seq data (GSE199230) via Seurat [32]. scRNA-seq data set was prepared using erythroid cells from the same donors (3/4) and using the same separation method (CD235a-positive magnetic separation) as was the NanoString assay in this paper and had high gene expression correlation with the NanoString assay (R = 0.8012). We tested the log10-transformed gene expression correlation using Pearson correlation in the GraphPad Prism 10.2.3. We subjected the expression matrix to a quality control procedure, found the most variable genes in expression for the merged matrix via *FindVariableFeatures*, performed SCTransform V2 data normalization using the variable features on the merged data, performed PCA (principal component analysis) dimensionality reduction on the normalized data, performed the UMAP dimensionality using 20 principal components. Then, we found Erythroid cell clusters that corresponded to the subsequent stages of erythroid cell differentiation. Clusters we identified using *ALAS2*, *CD36*, and *ITGA4* genes–*ALAS2*, *SCL25A37*, and *SCNA* gene expression gradually increased from the proerythroblasts stage and on, *CD36* and *ITGA4* gene gradually decreased from the proerythroblasts stage, *ARG1*+ Erythroid cells had the unique expression of *ARG1* gene. We also found two small subpopulations of erythroid cells– *DEFA3+* and Myeloid-like Erythroid cells. Then, we prepared SCT markers for the differential gene expression testing via *PrepSCTFindMarkers* and performed inter-cluster differential gene expression using the Wilcoxon test with biological and statistical significance criteria of log2(Fold Change) > 0.847 or log2(Fold Change) < −0.847 and q-value < 0.005 via *FindMarkers* and created the dot plot of the differentially expressed genes via *DotPlot* in Seurat.

For the comparative analysis with myeloid cells, we exported Myeloid-like Erythroid cell raw data, downloaded the gene expression matrix of the normal bone marrow mononuclear cells (*n* = 4) obtained with the same technology and gene panel (BD Rhapsody, Immune Response Panel) from the Gene Expression Omnibus (GSE261733, https://github.com/RIFCILab/ALL-BM-scMultiomics, accessed on 17.03.2024), exported pre-clustered Classic, Intermediate and Non-Classic Monocyte and Neutrophil raw data, merged the Myeloid-like Erythroid cell, Neutrophil, Classic, Intermediate and Non-Classic Monocyte raw data in Seurat via *Merge*, found the most variable genes in expression for the merged matrix via *FindVariableFeatures*, performed SCTransform V2 data normalization using the variable features on the merged data, prepared SCT markers for the differential gene expression testing via *PrepSCTFindMarkers*, performed differential gene expression testing using the Wilcoxon test with biological and statistical significance criteria of log2(Fold Change) > 0.847 or log2(Fold Change) < −0.847 and q-value < 0.005 via *FindMarkers*, created the dot plot of the differentially expressed genes via *DotPlot*.

## References

[1] Elahi S. Neglected cells: immunomodulatory roles of CD71+ erythroid cells. Trends in immunology. 2019 Mar 1;40(3):181–5. doi: 10.1016/j.it.2019.01.003 30718046

[2] The immunoregulatory function of peripheral blood CD71^+^ erythroid cells in systemic-onset juvenile idiopathic arthritis https://doi.org/10.1038%2Fs41598-021-93831-3 10.1038/s41598-021-93831-3 PMC8277864 34257378

[3] Zhao GJ, Jiang DW, Cai WC, Chen XY, Dong W, Chen LW, et al. CD71+ Erythroid Cell Expansion in Adult Sepsis: Potential Causes and Role in Prognosis and Nosocomial Infection Prediction. Frontiers in immunology. 2022 Feb 18;13:830025. doi: 10.3389/fimmu.2022.830025 35251018 PMC8896534

[4] Perik-Zavodskaia O, Perik-Zavodskii R, Nazarov K, Volynets M, Alrhmoun S, Shevchenko J, et al. Murine Bone Marrow Erythroid Cells Have Two Branches of Differentiation Defined by the Presence of CD45 and a Different Immune Transcriptome Than Fetal Liver Erythroid Cells. International Journal of Molecular Sciences. 2023 Oct 30;24(21):15752. doi: 10.3390/ijms242115752 37958735 PMC10650492

[5] Grzywa TM, Czubak K, Sidor K, Pilch Z, Bragiel-Pieczonka A, Hoser G, et al. Immunoregulatory CD71+ Erythroid Cells (Erythroid cells) Expand in Multiple Myeloma and Impair Control of L. Monocytogenes Infection. Blood. 2022 Nov 15;140(Supplement 1):12538–9. doi: 10.1182/blood-2022-164577

[6] Bozorgmehr N, Okoye I, Mashhouri S, Lu J, Koleva P, Walker J, et al. CD71+ erythroid cells suppress T-cell effector functions and predict immunotherapy outcomes in patients with virus-associated solid tumors. Journal for Immunotherapy of Cancer. 2023;11(5). doi: 10.1136/jitc-2022-006595 37236637 PMC10230995

[7] Rajashekaraiah V, Pallavi M, Choudhary A, Bhat C, Banerjee P, Laavanyaa S, et al. Reactive Oxygen Species and Antioxidant Interactions in Erythrocytes. 10.5772/intechopen.107544

[8] Elahi S, Vega-López MA, Herman-Miguel V, Ramírez-Estudillo C, Mancilla-Ramirez J, Motyka B, et al. CD71+ erythroid cells in human neonates exhibit immunosuppressive properties and compromise immune response against systemic infection in neonatal mice. Frontiers in immunology. 2020 Nov 24;11:597433. doi: 10.3389/fimmu.2020.597433 33329589 PMC7732591

[9] Perik-Zavodskii R, Perik-Zavodskaia O, Shevchenko J, Denisova V, Alrhmoun S, Volynets M, et al. Immune Transcriptome Study of Human Nucleated Erythroid Cells from Different Tissues by Single-Cell RNA-Sequencing. Cells. 2022 Nov 9;11(22):3537. doi: 10.3390/cells11223537 36428967 PMC9688070

[10] Grzywa TM, Sosnowska A, Rydzynska Z, Lazniewski M, Plewczynski D, Klicka K, et al. Potent but transient immunosuppression of T-cells is a general feature of CD71+ erythroid cells. Communications Biology. 2021 Dec 10;4(1):1384. doi: 10.1038/s42003-021-02914-4 34893694 PMC8664950

[11] Elahi S, Ertelt JM, Kinder JM, Jiang TT, Zhang X, Xin L, et al. Immunosuppressive CD71+ erythroid cells compromise neonatal host defence against infection. Nature. 2013 Dec 5;504(7478):158–62. doi: 10.1038/nature12675 24196717 PMC3979598

[12] Nazarov K, Perik-Zavodskii R, Perik-Zavodskaia O, Alrhmoun S, Volynets M, Shevchenko J, et al. Murine Placental Erythroid Cells Are Mainly Represented by CD45+ Immunosuppressive Erythroid Cells and Secrete CXCL1, CCL2, CCL3 and CCL4 Chemokines. International Journal of Molecular Sciences. 2023 May 1;24(9):8130. doi: 10.3390/ijms24098130 37175837 PMC10179598

[13] Sennikov SV, Krysov SV, Injelevskaya TV, Silkov AN, Kozlov VA. Production of cytokines by immature erythroid cells derived from human embryonic liver. European cytokine network. 2001 Jun 8;12(2):274–9. 11399516

[14] Sennikov SV, Injelevskaya TV, Krysov SV, Silkov AN, Kovinev IB, Dyachkova NJ, et al. Production of hemo-and immunoregulatory cytokines by erythroblast antigen+ and glycophorin A+ cells from human bone marrow. BMC Cell Biology. 2004 Dec;5(1):1–6. doi: 10.1186/1471-2121-5-39 15488155 PMC524510

[15] Seledtsov VI, Seledtsova GV, Samarin DM, Taraban VY, Sennikov SV, Kozlov VA. Characterization of erythroid cell-derived natural suppressor activity. Immunobiology. 1998 Feb 1;198(4):361–74. doi: 10.1016/S0171-2985(98)80045-4 9562862

[16] Sennikov SV, Eremina LV, Samarin DM, Avdeev IV, Kozlov VA. Cytokine gene expression in erythroid cells. European cytokine network. 1996 Dec 1;7(4):771–4. 9010680

[17] Sennikov SV, Krysov SV, Silkov AN, Injelevskaya TV, Kozlov VA. Production of IL-10, TNF-α, IFN-γ, TGF-β1 by different populations of erythroid cells derived from human embryonal liver. Cytokine. 2002 Feb 1;17(4):221–5. doi: 10.1006/cyto.2001.0975 11991675

[18] Long H, Jia Q, Wang L, Fang W, Wang Z, Jiang T, et al. Tumor-induced erythroid precursor-differentiated myeloid cells mediate immunosuppression and curtail anti-PD-1/PD-L1 treatment efficacy. Cancer cell. 2022 Jun 13;40(6):674–93. doi: 10.1016/j.ccell.2022.04.018 35594863

[19] Motamedi M, Xu L, Elahi S. Correlation of transferrin receptor (CD71) with Ki67 expression on stimulated human and mouse T cells: The kinetics of expression of T cell activation markers. Journal of immunological methods. 2016 Oct 1;437:43–52. doi: 10.1016/j.jim.2016.08.002 27555239

[20] Badal D, Dayal D, Singh G, Sachdeva N. Role of DNA-LL37 complexes in the activation of plasmacytoid dendritic cells and monocytes in subjects with type 1 diabetes. Scientific Reports. 2020 Jun 1;10(1):8896. doi: 10.1038/s41598-020-65851-y 32483133 PMC7264208

[21] Liu Q, Wang M, Hu Y, Xing H, Chen X, Zhang Y, et al. Significance of CD71 expression by flow cytometry in diagnosis of acute leukemia. Leukemia & lymphoma. 2014 Apr 1;55(4):892–8. doi: 10.3109/10428194.2013.819100 23962073

[22] Gahmberg CG, Ekblom M, Andersson LC. Differentiation of human erythroid cells is associated with increased O-glycosylation of the major sialoglycoprotein, glycophorin A. Proceedings of the National Academy of Sciences. 1984 Nov;81(21):6752–6. doi: 10.1073/pnas.81.21.6752 6387712 PMC392009

[23] Zhu F, Shi L, Li H, Eksi R, Engel JD, Guan Y. Modeling dynamic functional relationship networks and application to ex vivo human erythroid differentiation. Bioinformatics. 2014 Dec 1;30(23):3325–33. doi: 10.1093/bioinformatics/btu542 25115705 PMC4296147

[24] Sadlon TJ, Dell’Oso T, Surinya KH, May BK. Regulation of erythroid 5-aminolevulinate synthase expression during erythropoiesis. The international journal of biochemistry & cell biology. 1999 Oct 1;31(10):1153–67. doi: 10.1016/s1357-2725(99)00073-4 10582344

[25] Chen W, Paradkar PN, Li L, Pierce EL, Langer NB, Takahashi-Makise N, et al. Abcb10 physically interacts with mitoferrin-1 (Slc25a37) to enhance its stability and function in the erythroid mitochondria. Proceedings of the National Academy of Sciences. 2009 Sep 22;106(38):16263–8. doi: 10.1073/pnas.0904519106 19805291 PMC2752562

[26] McKenna E, Mhaonaigh AU, Wubben R, Dwivedi A, Hurley T, Kelly LA, et al. Neutrophils: need for standardized nomenclature. Frontiers in Immunology. 2021 Apr 15;12:602963. doi: 10.3389/fimmu.2021.602963 33936029 PMC8081893

[27] Kapellos TS, Bonaguro L, Gemünd I, Reusch N, Saglam A, Hinkley ER, et al. Human monocyte subsets and phenotypes in major chronic inflammatory diseases. Frontiers in immunology. 2019 Aug 30;10:2035. doi: 10.3389/fimmu.2019.02035 31543877 PMC6728754

[28] Bock AM, Cao Q, Ferrieri P, Young JA, Weisdorf DJ. Bacteremia in blood or marrow transplantation patients: clinical risk factors for infection and emerging antibiotic resistance. Biology of blood and marrow transplantation. 2013 Jan 1;19(1):102–8. doi: 10.1016/j.bbmt.2012.08.016 22940054

[29] Schajnovitz A, Itkin T, D’uva G, Kalinkovich A, Golan K, Ludin A, et al. CXCL12 secretion by bone marrow stromal cells is dependent on cell contact and mediated by connexin-43 and connexin-45 gap junctions. Nature immunology. 2011 May;12(5):391–8. doi: 10.1038/ni.2017 21441933

[30] reneshbedre/bioinfokit: Bioinformatics data analysis and visualization toolkit https://zenodo.org/doi/10.5281/zenodo.3698145

[31] Fang Z, Liu X, Peltz G. GSEApy: a comprehensive package for performing gene set enrichment analysis in Python. Bioinformatics. 2023 Jan 1;39(1):btac757. doi: 10.1093/bioinformatics/btac757 36426870 PMC9805564

[32] Hao Y, Hao S, Andersen-Nissen E, Mauck WM, Zheng S, Butler A, et al. Integrated analysis of multimodal single-cell data. Cell. 2021 Jun 24;184(13):3573–87. doi: 10.1016/j.cell.2021.04.048 34062119 PMC8238499

